# Laparoscopic and robotic ventral rectopexy for posterior compartment disorders: a multicentric study in a gynecological setting

**DOI:** 10.1007/s10151-026-03365-x

**Published:** 2026-06-09

**Authors:** Giovanni Panico, Sara Mastrovito, Tomaso Melocchi, Giuseppe Campagna, Alfredo Ercoli, Chrystele Rubod, Michel Cosson, Marine Lallemant, Alessandro Ferdinando Ruffolo

**Affiliations:** 1 Department of Woman and Child’s Health and Public Health, Division of Urogynecology and Reconstructive Surgery of Pelvic Floor, Fondazione Policlinico Universitario Agostino Gemelli IRCCS, Rome, Italy; 2 Department of Gynecology, CHU Lille, 59000 Lille, France; 3 Department of Gynecology, Fondazione IRCCS San Gerardo Dei Tintori, Monza, Italy; 4 UOC of Gynecological Surgery and Urogynecology, Centre of Excellence Women and Childbirth, Ospedale Isola Tiberina – Gemelli Isola, Rome, Italy; 5 Department of Human Pathology in Adult and Childhood “G. Barresi”, Unit of Gynecology and Obstetrics, University of Messina, Messina, Italy; 6 Faculté de Médecine, University of Lille, 59000 Lille, France; 7 UMR 9013 - LaMcube - Laboratoire de Mécanique, Multiphysique, Multiéchelle, University of Lille, CNRS, Centrale Lille, 59000 Lille, France; 8 University of Lille, CHU Lille, ULR 2694 - METRICS, 59000 Lille, France

**Keywords:** Ventral rectopexy, Pelvic organ prolapse, Enterocele, Rectal prolapse, Constipation, Anal incontinence

## Abstract

**Introduction:**

This study aimed to evaluate postoperative outcomes of minimally invasive ventral rectopexy (VRP) performed for posterior compartment defects in a gynecological setting, according to laparoscopic (L-VRP) or robotic (R-VRP) approaches.

**Methods:**

Women with symptomatic posterior compartment defects, including enterocele, rectal prolapse, and rectocele, who underwent L-VRP or R-VRP, were retrospectively analyzed. Primary outcome was the evaluation of functional outcomes at ≥ 6-month follow-up; secondary outcomes included anatomical results, peri- and postoperative complications, and predictors of recurrence, postoperative constipation, and anal incontinence.

**Results:**

A total of 102 women were included, 60 in the L-VRP group and 42 in the R-VRP group. Anterior sacrocolpopexy was performed in 70.6% of women (72/102). At a mean of 18-month follow-up, anorectal symptoms rate, constipation [L-VRP (17.7 ± 5.5) versus (2.5 ± 3.6): *p* < 0.001; R-VRP (15.2 ± 5.4) versus (2.6 ± 4.6): *p* < 0.001], and incontinence [L-VRP (11.6 ± 4.6) versus (1.1 ± 3.1): *p* < 0.001; R-VRP (10.7 ± 5.1) versus (1.2 ± 2.2): *p* < 0.001] Wexner scores significantly improved without difference according to the surgical route (*p* > 0.05). Posterior prolapse relapse was 1.0% (1/101), without difference between groups [L-VRP (1.7%;1/60) versus R-VRP (0%;0/41); *p* = 0.99]. Other anatomical and functional outcomes did not significantly differ between groups. One woman in L-VRP group (1.0%; 1/101) required the resection of the mesh for infection of the implanted material. No predictive factors emerged at multivariate analysis.

**Conclusions:**

Minimally invasive VRP was associated with significant short- to mid-term anatomical and functional improvement and a low rate of perioperative complications. No statistically significant differences were observed between laparoscopic and robotic approaches; however, the study was not powered to demonstrate equivalence, and longer follow-up is required to assess durability and delayed mesh-related complications.

**Trial registration:**

DIPUSVSP-27–07-2087 retrospectively registered.

## Introduction

Posterior compartment disorders, encompassing enterocele and rectal prolapse (RP), represent a significant subset of pelvic floor dysfunction (PFD) frequently encountered in gynecological practice. Multicompartmental involvement is common, with posterior prolapse identified in a notable proportion of women presenting with pelvic organ prolapse (POP) [1, 2], particularly among the elderly [3].

The anatomical spectrum of posterior compartment disorders includes rectocele, enterocele, and RP, each posing distinct diagnostic and therapeutic challenges. Enterocele is defined by the herniation of a peritoneal sac, often containing small bowel, between the rectum and vagina, whereas RP involves the full-thickness protrusion of the rectal wall through the anal canal, sometimes associated with peritoneal descent [4–7].

Risk factors for these conditions include advanced age, multiparity, obesity, and prior hysterectomy, which alters apical support and predisposes to enterocele formation [8–10].

Clinically, patients may report pelvic pressure, a sensation of vaginal bulging, pelvic pain (particularly with enterocele due to mesenteric traction), and various anorectal symptoms such as fecal/anal incontinence or obstructed defecation [11, 12].

Diagnostic imaging, including dynamic pelvic magnetic resonance imaging (dMRI) and defecography, is essential for accurate characterization, though cost and accessibility often limit their widespread use [13–15].

Ventral rectopexy (VRP) has emerged as the preferred surgical technique for addressing posterior compartment prolapse, particularly in cases of enterocele and RP [16–18]. By providing anterior suspension of the rectum and reinforcing the pelvic floor, VRP aims to restore normal anatomy, improve functional outcomes, and reduce recurrence [19, 20].

The dissection required for VRP closely parallels that performed during sacrocolpopexy (SCP), involving exposure of the promontory, dissection along the anterior rectal wall, and fixation to the longitudinal ligament of the sacrum. This shared anatomical and technical approach facilitates the integration of VRP into gynecologic surgical practice, enabling gynecologic surgeons, already proficient in SCP, to safely perform the procedure when clinically indicated.

Both laparoscopic and robotic approaches are currently employed, with the robotic platform potentially offering technical advantages in complex dissections and suturing within confined pelvic spaces.

While both laparoscopic and robotic techniques are employed for VRP, most published series to date originate from colorectal surgical settings, often including mixed female–male populations [21].

Few studies have evaluated outcomes in women operated within a gynecologic context, and the overall feasibility of integrating VRP into routine gynecologic surgical practice, where the indication typically arises from complex multicompartmental pelvic floor dysfunction involving the posterior, apical, and anterior compartments.

The primary aim of this multicentric study is therefore to assess and compare the functional outcomes of laparoscopic versus robotic VRP performed exclusively in female patients managed within gynecologic departments for posterior compartment disorders. Secondary objectives include evaluating perioperative safety, postoperative anatomical outcomes, and risk factors for anatomical recurrence and postoperative anorectal symptoms.

## Methods

This was a retrospective multicentric study conducted between February 2021 and February 2025 at the gynecology departments of Jeanne de Flandre Hospital, Lille, France, and of Fondazione Policlinico Universitario Agostino Gemelli–IRCCS, Rome, Italy.

### Human ethics and consent to participate declarations

The study was performed according to the Declaration of Helsinki and received approval from the institutional review board (DIPUSVSP-27–07-2087). All participants provided informed consent for the use of their clinical data for research purposes.

### Study population and inclusion criteria

Women with symptomatic posterior pelvic floor disorder who underwent minimally invasive VRP were included. Posterior pelvic floor disorders considered eligible for VRP were enterocele or RP. Additional anterior SCP was performed in women affected by anterior and/or apical POP.

### Study procedure

Preoperative evaluation included the collection of medical history, assessment of PFD symptoms, and a physical examination performed by a trained urogynecologist. The terminology used to describe PFD symptoms was in accordance with the standardization document of the IUGA/ICS [22]. Functional pelvic floor and anorectal symptoms were retrospectively extracted from standardized clinical consultation records. Constipation and fecal incontinence were assessed using validated Wexner scoring systems. Physical examination was performed in the lithotomy position, both at rest and during straining. All women presenting with anorectal symptoms and/or symptomatic posterior vaginal wall prolapse with suspicion of enterocele or RP underwent defecography or dMRI. The grade of vaginal prolapse was recorded according to the Pelvic Organ Prolapse-Quantification (POP-Q) system [6], while the grade of RP was classified by the Oxford Prolapse Grading System [23]. A significant RP was defined as stage ≥ 3 [24].

The indication for VRP was determined in a multidisciplinary setting involving gynecologists, colorectal surgeons, gastroenterologists, urologists, radiologists, and pain specialists.

The study population was divided into two subgroups according to the surgical route: robotic (R-VRP) or laparoscopic (L-VRP). No predefined clinical or anatomical criteria were used to select patients for laparoscopic or robotic ventral rectopexy; the surgical approach was chosen according to robotic platform availability.

### Perioperative data were recorded

Patients were advised to refrain from heavy lifting and sexual intercourse for 6–8 weeks after surgery.

Peri- and postoperative complications were assessed using the Clavien–Dindo classification [25].

At postoperative follow-up beyond 6 months, PFD symptoms and a physical examination were reassessed. Posterior vaginal wall recurrence was defined as ≥ II stage according to the POP-Q system. Apical or anterior vaginal relapse was defined as ≥ II stage.

The Wexner constipation and incontinence questionnaires were systematically administered at baseline and at follow-up, and prospectively collected [26, 27].

The primary outcome was to evaluate the functional anorectal outcomes in women undergoing minimally invasive VRP and to compare results according to the surgical route.

Secondary outcomes included the evaluation of anatomical posterior compartment outcomes, other compartment relapses, intra- and postoperative complications according to the surgical route, preoperative risk factors for anatomical recurrence, postoperative constipation symptoms, and anal incontinence. Posterior compartment defect characteristics, including presence of enterocele, rectal prolapse (Oxford stage), and rectocele, were included in the univariate and multivariate analyses.

### Surgical procedure

VRP was performed using either conventional laparoscopy or robot-assisted laparoscopy under general anesthesia. The procedure began with a thorough exploration of the abdominal and pelvic cavities to assess the anatomy and identify any unexpected findings. The longitudinal vertebral ligament overlying the sacral promontory was exposed by dissecting parallel to the right common iliac artery, with particular care taken to avoid injury to the right hypogastric nerve at the pelvic inlet. The peritoneum was then incised along the right pelvic wall and extended down to the pouch of Douglas. This pouch was opened between the left and right uterosacral ligaments, allowing access to the rectovaginal space, which was carefully dissected along the posterior vaginal wall. The dissection extended caudally to the perineal body and laterally to the lateral ligaments.

Once the dissection was complete, a polypropylene mesh, tailored to fit the anatomical area, was placed and secured to the anterior aspect of the rectum at the seromuscular layer using six 2–0 or 3–0 non-absorbable sutures. The first suture was always placed at the most distal part of the anterior rectal wall, close to the perineal body. Posterior vaginal fornix was elevated and sutured to the anterior rectal wall to correct vaginal vault prolapse, following the technique described by D’Hoore [28].

When anterior SCP was performed concurrently with VRP, the vesicovaginal space was dissected up to the dorsal end of the bladder trigone. A second synthetic mesh, shaped to cover the entire dissected area, was then fixed to the anterior vaginal wall and the dorsal part of the uterine cervix or vaginal vault with three 2–0 or 3–0 absorbable monofilament sutures. Both meshes were then directed toward the sacral promontory and anchored to the previously exposed longitudinal vertebral ligament using a size 0 non-absorbable suture. Finally, the peritoneum was closed over the mesh to ensure complete coverage and reduce the risk of adhesions. In cases where hysterectomy was indicated, a supracervical hysterectomy with bilateral salpingectomy or salpingo-oophorectomy was performed to preserve vaginal integrity and minimize mesh-related complications.

### Statistical analysis

Statistical analysis was performed using *Jamovi* software version 2.3.28.0 (Sydney, Australia). Continuous variables were reported as mean ± standard deviation (SD) or as median ± interquartile range, as appropriate.

The Kolmogorov–Smirnov test was used to analyze the normal distribution of variables (*p* > 0.05).

The statistical significance of difference in distribution was tested with Pearson chi-squared test for independent samples, the McNemar test for paired samples, and the exact Fisher test for categorical variables as appropriate. For continuous variables the *t*-test for independent or paired samples, the Mann–Whitney *U*-test for intergroup differences, and the Wilcoxon rank sum test for intragroup differences were adopted as appropriate. The statistical analysis was conducted at 95% confidence level.

Univariate and multivariate analyses were applied to variables. Significant association was considered at 95% confidence level.

## Results

A total of 102 women affected by a posterior compartment defect who underwent endoscopic VRP were included into this study. The baseline clinical characteristics of the overall study population are presented in Table 1. Notably, 52.0% (53/102) of patients had at least one previous prolapse surgery and 37.3% (38/102) had a previous hysterectomy. Previous lateral suspension was reported in 13.7% of the population (14/102). Women submitted to R-VRP in comparison with L-VRP presented a higher rate of hysterectomy (23.3% versus 57.1%; *p* < 0.001) and of previous posterior vaginal repair (3.3% versus 19.0%; *p* = 0.01), but a lower rate of lateral suspension (23.3% versus 0%; *p* < 0.001). No other difference in baseline characteristics was highlighted between groups.

**Table 1:** General characteristics of the study population

Characteristics	Overall (*n* = 102)	Laparoscopic (*n* = 60)	Robotic (*n* = 42)	*p*-Value
Age, year, mean ± SD	62.9 ± 11.0	62.4 ± 11.1	63.6 ± 10.9	0.53
BMI, kg/m^2^, mean ± SD	25.2 ± 4.4	25.7 ± 4.6	24.5 ± 3.8	0.31
Parity, median (IQR)	2 (1.2–3.0)	2.5 (2–3.2)	2 (1–2)	**0.001**
Cesarean section, *n* (%)	5 (4.9)	2 (3.3)	3 (7.1)	0.40
Smoking habit, *n* (%)	19 (18.6)	11 (18.3)	8 (19.0)	0.93
Menopause, *n* (%)	89 (87.3)	51 (85.0)	38 (90.5)	0.55
Instrumental delivery, *n* (%)	19 (18.6)	12 (20.0)	7 (16.7)	0.80
Episiotomy, *n* (%)	23 (22.5)	15 (25.0)	8 (19.0)	0.48
OASIS, *n* (%)	4 (3.9)	3 (5.0)	1 (2.4)	0.64
Previous hysterectomy, *n* (%)	38 (37.3)	14 (23.3)	24 (57.1)	** < 0.001**
Previous POP surgery, *n* (%)	53 (52.0)	33 (55.0)	20 (47.6)	0.46
Anterior vaginal repair, *n* (%)	15 (14.7)	9 (15.0)	6 (14.3)	0.92
NTR	11 (10.8)	6 (10.0)	5 (11.9)	
TVM	5 (4.9)	4 (3.9)	1 (2.4)	
Apical vaginal repair, *n* (%)	16 (15.7)	8 (13.3)	8 (19.0)	0.43
NTR	15 (14.7)	7 (11.7)	8 (19.0)	
TVM	3 (0)	3 (5.0)	0 (0)	
Posterior vaginal repair, *n* (%)	10 (9.8)	2 (3.3)	8 (19.0)	**0.01**
NTR	10 (9.8)	2 (3.3)	8 (19.0)	
TVM	0 (0)	0 (0)	0 (0)	
Anterior sacrocolpopexy, *n* (%)	13 (12.7)	5 (8.3)	8 (19.0)	0.13
Posterior sacrocolpopexy, *n* (%)	6 (5.9)	1 (1.7)	5 (11.9)	0.08
Ventral rectopexy, *n* (%)	4 (3.9)	2 (3.3)	2 (4.8)	0.99
Lateral culposuspension, *n* (%)	14 (13.7)	14 (23.3)	0 (0)	** < 0.001**
SUI surgery, *n* (%)	14 (13.7)	6 (10.0)	8 (19.0)	0.25

*BMI* body mass index, *IQR* interquartile range, *NTV* native tissue repair, *OASIS* obstetric anal sphincter injuries, *POP* pelvic organ prolapse, *RP* rectal prolapse, *SD* standard deviation, *SUI* stress urinary incontinence, *TVM* transvaginal mesh

Bold denotes statistically significant value (*p*<0.05)

Preoperative assessment of posterior compartment defect through defecography or dMRI is presented in Table 2. Preoperative analysis showed that 68.6% (70/102) of women presented an enterocele, while 39.2% (40/102) had an associated rectocele. Moreover, 37.3% (38/102) of patients had a significant RP, with a variable distribution across different stages according to the Oxford Prolapse Grading System. There was no difference in posterior compartment defect distribution in women submitted to L-VRP or R-VRP.

**Table 2:** Posterior compartment defect at MR/defecography

Posterior compartment defect at dMRI/defecography	Overall (*n* = 102)	Laparoscopic (*n* = 60)	Robotic (*n* = 42)	*p*-Value
Enterocele	70 (68.6)	41 (68.3)	29 (69.0)	0.94
Rectocele	40 (39.2)	18 (30.0)	22 (52.4)	**0.02**
Clinically significant rectal prolapse^a^	38 (37.3)	26 (43.3)	12 (28.6)	0.13
Stage 3	2/38 (5.3)	1/26 (3.8)	1/12 (8.3)	
Stage 4	14/38 (36.8)	8/26 (30.8)	6/12 (50.0)	
Stage 5	22/38 (57.9)	17/26 (65.4)	5/12 (41.7)	

Bold denotes statistically significant value (*p*<0.05)

^a^According to the Oxford Prolapse Grading System

Additional surgeries at time of VRP and intra- and perioperative data are reported in Table 3; 70.6% of women (72/102) underwent concomitant anterior SCP for anterior/apical POP.

**Table 3:** Intra- and perioperative data

Intra- and perioperative data	Overall (*n* = 102)	Laparoscopic (*n* = 60)	Robotic (*n* = 42)	*p*-Value
Surgeon experience, *n* (%):				
Senior	74 (72.5)	45 (75.0)	29 (69.0)	0.50
Fellow	28 (27.5)	15 (25.0)	13 (31.0)	
Subtotal hysterectomy, *n* (%)	52 (51.0)	39 (65.0)	13 (31.0)	** < 0.001**
Anterior sacrocolpopexy, *n* (%)	72 (70.6)	50 (83.3)	22 (52.4)	** < 0.001**
Other concomitant surgeries, *n* (%):	3 (3.0)	2 (3.3)	1 (2.4)	0.99
RP-TVT	1 (1.0)	1 (1.7)	0 (0)	
UBA	1 (1.0)	1 (1.7)	0 (0)	
Ablation of a vaginal synechia	1 (1.0)	0 (0)	1 (2.4)	
Operative time, min, mean ± SD	162 ± 50.7	158 ± 50.2	169 ± 52.1	0.42
Intraoperative complication, *n* (%):	5 (5.0)	3 (5.0)	2 (4.8)	0.99
Conversion to laparotomy for inaccessible promontory	3 (3.0)	2 (3.3)	1 (2.4)	
Anterior vaginal wall opening	1 (1.0)	1 (1.7)	0 (0)	
Interruption of the procedure	1 (1.0)	0 (0)	1 (2.4)	
Peri- and postoperative complication (Clavien–Dindo System ≥ 3), *n* (%)				
Mesh infection requiring resection	1 (1.0)	1 (1.7)	0 (0)	0.99
Hospitalization stays, median (IQR)	2 (2–2)	2 (1–2)	2 (2–2)	0.11

*IQR* interquartile range, *RP-TVT* retropubic tension-free vaginal tape, *RP* rectal prolapse, *SD* standard deviation, *UBA* urethral bulking agent

Bold denotes statistically significant value (*p*<0.05)

Laparotomic conversion was required only in three cases (3.0%; 3/102) for impossibility to reach the promontory in relation to abdominopelvic adhesions. One patient (1%; 1/102) had the surgical procedure interrupted due to abdominal and pelvic adhesions. No difference in intra- and perioperative outcome was found in the two subgroups.

Pre- and postoperative PFD symptoms and anatomical results at follow-up are presented in Table 4; 80.2% of the study population presented with anorectal symptoms (81/101). However, there were no differences in specific anorectal symptoms at baseline, except for a higher prevalence of feeling of incomplete bowel evacuation (58.3% versus 36.6%; *p* = 0.03) and anal bloating (21.7% versus 4.9%; *p* = 0.02) in the L-VRP group. Considering the severity of anorectal symptoms at baseline, constipation and incontinence Wexner score did not differ between L-VRP and R-VRP groups. At a mean of 18-month follow-up, almost all anorectal symptoms improved in the general population and in subgroups. No difference in prevalence of anorectal symptoms between subgroups was reported at follow-up. The constipation and incontinence Wexner score showed postoperative reduction in the study population, without differences between subgroups.

**Table 4:** Ventral rectopexy outcomes in women with enterocele and impact of concurrent rectal prolapse

Outcomes	Overall (*n* = 101)	Laparoscopic (*n* = 60)	Robotic (*n* = 41)	*p*-Value
SUI, *n* (%)				
Preop	32 (31.7)	19 (31.7)	13 (31.7)	0.99
Postop	26 (25.7)	16 (26.7)	10 (24.4)	0.80
* p-*Value	0.24	0.40	0.40	
Urinary urgency, *n* (%)				
Preop	44 (43.6)	26 (43.3)	18 (43.9)	0.95
Postop	18 (17.8)	8 (13.3)	10 (24.4)	0.15
* p-*Value	** < 0.001**	** < 0.001**	**0.03**	
UUI, *n* (%)				
Preop	25 (24.8)	17 (28.3)	8 (19.5)	0.31
Postop	17 (16.8)	8 (13.3)	9 (22.0)	0.27
* p-*Value	0.09	**0.03**	0.70	
Voiding difficulty, *n* (%)				
Preop	35 (34.7)	19 (31.7)	16 (39.9)	0.44
Postop	6 (5.9)	5 (8.3)	1 (2.4)	0.40
* p-*Value	** < 0.001**	**0.002**	** < 0.001**	
Overall anorectal symptoms, *n* (%)				
Preop	81 (80.2)	52 (86.7)	29 (70.7)	0.05
Postop	26 (25.7)	14 (23.3)	12 (29.3)	0.64
* p-*Value	** < 0.001**	** < 0.001**	** < 0.001**	
Constipation, *n* (%)				
Preop	53 (52.5)	35 (58.3)	18 (43.9)	0.15
Postop	17 (16.8)	10 (16.7)	7 (17.1)	0.96
* p-*Value	** < 0.001**	** < 0.001**	**0.005**	
Feeling of incomplete bowel evacuation, *n* (%)				
Preop	50 (49.5)	35 (58.3)	15 (36.6)	**0.03**
Postop	8 (7.9)	4 (6.7)	4 (9.8)	0.71
* p-*Value	** < 0.001**	** < 0.001**	**0.005**	
Pain at defecation, *n* (%)				
Preop	17 (16.8)	12 (20.0)	5 (12.2)	0.30
Postop	4 (4.0)	3 (5.0)	1 (2.4)	0.64
*p-*Value	**0.002**	**0.01**	**0.04**	
Splinting, *n* (%)				
Preop	25 (24.8)	13 (21.7)	12 (29.3)	0.38
Postop	0 (0)	0 (0)	0 (0)	0.99
* p-*Value	** < 0.001**	** < 0.001**	** < 0.001**	
Straining to defecate, *n* (%)				
Preop	41 (40.6)	25 (41.7)	16 (39.0)	0.79
Postop	4 (4.0)	3 (5.0)	1 (2.4)	0.64
* p-*Value	** < 0.001**	** < 0.001**	** < 0.001**	
Bloating, *n* (%)				
Preop	15 (14.9)	13 (21.7)	2 (4.9)	**0.02**
Postop	5 (5.0)	4 (6.7)	1 (2.4)	0.64
* p-*Value	**0.008**	**0.01**	0.31	
Flatus incontinence, *n* (%)				
Preop	21 (20.8)	13 (21.7)	8 (19.5)	0.79
Postop	5 (5.0)	3 (5.0)	2 (4.9)	0.99
* p-*Value	** < 0.001**	**0.004**	**0.03**	
Fecal incontinence, *n* (%)				
Preop	24 (23.8)	14 (23.3)	10 (24.4)	0.90
Postop	8 (7.9)	4 (6.7)	4 (9.8)	0.71
* p-*Value	**0.001**	**0.01**	**0.03**	
Wexner constipation, mean ± SD*:				
Preop	17.2 ± 5.6	17.7 ± 5.5	15.2 ± 5.4	0.11
Postop	2.6 ± 3.8	2.5 ± 3.6	2.6 ± 4.6	0.96
* p-*Value	** < 0.001**	** < 0.001**	** < 0.001**	
Wexner incontinence, mean ± SD^§^:				
Preop	11.2 ± 4.7	11.6 ± 4.6	10.7 ± 5.1	0.66
Postop	1.1 ± 2.1	1.1 ± 2.1	1.2 ± 2.2	0.90
* p-*Value	** < 0.001**	** < 0.001**	** < 0.001**	
Anal bulging, *n* (%)				
Preop	31 (30.7)	18 (30.0)	13 (31.7)	0.85
Postop	2 (2.0)	2 (3.3)	0 (0)	0.51
* p-*Value	** < 0.001**	** < 0.001**	** < 0.001**	
Vaginal bulging				
Preop	79 (78.2)	51 (85.0)	28 (68.3)	0.05
Postop	0 (0)	0 (0)	0 (0)	0.99
* p-*Value	** < 0.001**	** < 0.001**	** < 0.001**	
Ba ≥ −1 cm, *n* (%)				
Preop	56 (55.4)	36 (60.0)	20 (48.8)	0.26
Postop	9 (8.9)	6 (10.0)	3 (7.3)	0.73
* p-*Value	** < 0.001**	** < 0.001**	** < 0.001**	
Apical POP ≥ 2 stage, *n* (%)				
Preop	57 (56.4)	40 (66.7)	17 (41.5)	**0.01**
Postop	1 (1.0)	0 (0)	1 (2.4)	0.41
* p-*Value	** < 0.001**	** < 0.001**	** < 0.001**	
Bp ≥ −1 cm, *n* (%)				
Preop	67 (66.3)	41 (68.3)	26 (63.4)	0.60
Postop	1 (1.0)	1 (1.7)	0 (0)	0.99
* p-*Value	** < 0.001**	** < 0.001**	** < 0.001**	
Reoperation, *n* (%)	8 (7.9)	6 (10.0)	2 (4.9)	0.47
TOT	6 (5.9)	6 (10.0)	0 (0)	
Resection of vaginal wire	1 (1.0)	0 (0)	1 (2.4)	
Redo SCP	1 (1.0)	0 (0)	1 (2.4)	
Follow-up, month, mean ± SD	18.5 ± 9.9	19.9 ± 10.9	16.5 ± 8.0	0.19

*POP* pelvic organ prolapse, *RP* rectal prolapse, *SD* standard deviation, *SUI* stress urinary incontinence, *UUI* urgency urinary incontinence

Bold denotes statistically significant value (*p*<0.05)

^*^Evaluated among women with preoperative constipation symptoms

^§^Evaluated among women with preoperative anal incontinence

At 18-month follow-up, one patient had anatomical posterior vaginal wall prolapse relapse in the laparoscopic group (1.7% versus 0%; *p* = 0.99). There was one reintervention for POP recurrence in the R-VRP group (0% versus 2.4%). No difference in other postoperative PFD symptoms and anatomical results was highlighted between women according to the surgical route.

Considering peri- and postoperative complications, one woman required a resection of the mesh for infection of the implanted material in the L-VRP group (1.7% versus 0%; *p* = 0.99) (Table 3).

It was not possible to assess risk factors for anatomical recurrence of posterior vaginal wall prolapse, given that only one recurrence was observed in the entire study population.

At univariate analysis, higher preoperative constipation and Wexner constipation and incontinence scores were associated with postoperative constipation (Table 5). Preoperative Wexner incontinence score correlated with postoperative anal incontinence (AI), while constipation variables showed an inverse relationship (Table 6). However, none of these associations remained significant in multivariate analysis. At multivariate analysis, the presence of enterocele, rectal prolapse, or rectocele was not independently associated with postoperative constipation or anal incontinence. Given the limited number of postoperative events, these multivariable analyses should be considered exploratory and hypothesis generating.

**Table 5:** Predictive factors for postoperative constipation symptoms in women submitted to ventral rectopexy

	Univariate analysis		Multivariate analysis	
	Odds ratio	*p*-Value	Odds ratio	*p*-Value
Age, years	0.99 (0.95–1.03)	0.65	0.99 (0.94–1.04)	0.68
BMI, kg/m^2^	0.95 (0.85–1.08)	0.50	0.99 (0.87–1.13)	0.90
Parity	0.96 (0.67–1.36)	0.53	0.81 (0.50–1.31)	0.39
Preoperative enterocele	0.71 (0.26–1.94)	0.51	0.99 (0.27–3.64)	0.99
Preoperative rectal prolapse	0.98 (0.36–2.63)	0.98	0.43 (0.11–1.60)	0.21
Preoperative rectocele	0.83 (0.32–2.32)	0.72	1.21 (0.31–4.75)	0.78
Posterior prolapse stage at POP-Q	0.95 (0.67–1.35)	0.80	0.83 (0.50–1.37)	0.46
Robotic approach	1.01 (0.39–2.66)	0.97	0.77 (0.16–3.59)	0.74
Preoperative constipation symptoms	5.49 (1.19–25.22)	**0.03**	1.06 (0.05–20.71)	0.97
Preoperative anal incontinence symptoms	2.95 (1.10–7.84)	**0.03**	0.87 (0.05–12.95)	0.92
Preoperative Wexner constipation score	1.09 (1.02–1.16)	**0.006**	1.11 (0.97–1.26)	0.12
Preoperative Wexner anal incontinence score	1.10 (1.01–1.18)	**0.02**	1.15 (0.93–1.41)	0.17

*BMI* body mass index, *POP-Q* Pelvic Organ Prolapse-Quantification

Bold denotes statistically significant value (*p*<0.05)

**Table 6:** Predictive factors for postoperative anal incontinence in women submitted to ventral rectopexy

	Univariate analysis		Multivariate analysis	
	Odds ratio	*p*-Value	Odds ratio	*p*-Value
Age, years	0.99 (0.93–1.06)	0.86	1.03 (0.90–1.19)	0.06
BMI, kg/m^2^	0.77 (0.59–1.0)	0.10	0.46 (0.20–1.05)	0.06
Parity	1.09 (0.65–1.82)	0.72	0.83 (0.32–2.18)	0.71
Preoperative enterocele	1.36 (0.26–7.15)	0.71	8.99 (0.08–1040.82)	0.36
Preoperative rectal prolapse	1.18 (0.43–7.75)	0.42	0.52 (0.02–14.37)	0.70
Preoperative rectocele	0.48 (0.09–2.52)	0.39	8.03 (0.23–279.94)	0.25
Posterior prolapse stage at POP-Q	0.76 (0.43–1.33)	0.34	0.11 (0.01–1.38)	0.09
Robotic approach	0.19 (0.02–1.60)	0.13	0.01 (0.01–1.29)	0.06
Preoperative constipation symptoms	0.22 (0.05–0.99)	**0.04**	0.08 (0.01–199.47)	0.53
Preoperative anal incontinence symptoms	4.29 (0.95–19.27)	0.05	1.30 (0.01–4855.56)	0.94
Preoperative Wexner constipation score	0.90 (0.82–0.99)	**0.04**	0.83 (0.46–1.50)	0.54
Preoperative Wexner anal incontinence score	1.13 (1.02–1.26)	**0.02**	1.53 (0.70–3.33)	0.29

*BMI* body mass index, *POP-Q* Pelvic Organ Prolapse-Quantification

Bold denotes statistically significant value (*p*<0.05)

## Discussion

The findings of our study further support the effectiveness of VRP in the management of posterior compartment defects, as evidenced by an improvement in both anorectal and pelvic floor symptoms. VRP resulted in a low rate of anatomical recurrence in the posterior compartment at follow-up. Importantly, the anatomical success was consistent regardless of whether the procedure was performed laparoscopically or robotically, with no significant differences observed between surgical approaches. Restoration of normal pelvic organ anatomy was consistently achieved, leading to complete resolution of vaginal bulging in all patients and a significant reduction in anal bulging (30.7% versus 2.0%, *p* < 0.001), again with comparable outcomes across both surgical modalities. The d’Hoore technique for VRP, whether performed alone or in combination with anterior SCP, provides unique advantages through its dual action: it suspends the pelvic floor while integrating the rectum and vagina. This anatomic unification not only corrects rectal prolapse, but also effectively obliterates the rectovaginal space, thereby addressing enterocele and minimizing the risk of its recurrence. The combination of SCP with VRP has already been established as an effective approach for the management of multicompartmental POP, particularly in cases of RP associated with obstructed defecation [29] and enterocele [30]. This combined technique allows for simultaneous correction of both anterior/apical and posterior compartment defects, optimizing anatomical restoration and functional outcomes. In the specific context of enterocele, VRP has also been described by Mege et al. However, their reported failure rate of 31% may be attributed to the omission of a systematic vaginal suspension, a critical step that we have consistently incorporated in our methodology [18]. By systematically performing the vaginal suspension, we aim to stabilize the pelvic support structures and reduce the risk of recurrence, addressing a key limitation observed in previous studies. However, the very low posterior compartment recurrence rate observed in our cohort should be interpreted with caution. Anatomical recurrence was assessed clinically and no routine postoperative defecography or dynamic MRI was performed; therefore, subclinical or radiological recurrences may have been missed. In addition, the mean follow-up of 18.5 months primarily reflects early outcomes, whereas recurrence after VRP, particularly for enterocele, has been reported to occur at mid- to long-term follow-up, often beyond 2–3 years. Longer follow-up is required to evaluate recurrence patterns and the durability of anatomical correction.

Following surgery, VRP led to improvement in anorectal function. The prevalence of constipation, which affected 52.5% of patients preoperatively, dropped significantly to 16.8% after the intervention (*p* < 0.001), with similar benefits observed regardless of whether a laparoscopic or robotic approach was used. Likewise, the proportion of patients reporting a sensation of incomplete evacuation decreased from 49.5% to 7.9% (*p* < 0.001), indicating a substantial restoration of normal defecatory dynamics. These improvements were further substantiated by significant reductions in Wexner–Agachan scores within the study population. Our findings align with previous reports demonstrating that patients suffering from constipation or mixed symptoms of constipation and fecal incontinence often experience notable symptomatic relief after surgical correction of posterior compartment defects [29–32]. Additionally, a significant decrease in both fecal incontinence (from 23.8% to 7.9%, *p* < 0.001) and flatus incontinence (from 20.8% to 5.0%, *p* < 0.001) was observed, as reflected by improvements in the Jorge–Wexner score. These results are consistent with earlier studies that have shown enhanced sphincter control following correction of posterior compartment prolapse [33, 34]. While it is important to acknowledge that continence is inherently multifactorial, these improvements may be attributed not only to anatomical restoration and stabilization of the pelvic floor, but also to the specific surgical technique employed. Notably, VRP avoids posterolateral dissection of the rectum, thereby minimizing the risk of autonomic nerve injury, which could otherwise result in dysmotility and impaired evacuation. Subgroup analysis revealed no significant differences in the prevalence or severity of anorectal symptoms at follow-up on the basis of the surgical approach. This suggests that the key factor in functional recovery is the stabilization of the pelvic floor and rectal suspension achieved through VRP, which may be sufficient to restore appropriate anorectal function in many cases. Although no statistically significant differences were observed between laparoscopic and robotic VRP, this finding should not be interpreted as evidence of equivalence. The retrospective design, baseline differences between groups, limited sample size, and low number of outcome events preclude firm comparative conclusions.

Our findings further underscore the benefits of combining SCP with VRP. Consistent with previous reports, this integrated surgical approach allows for the effective correction of multicompartmental prolapse, resulting in significant improvements not only in anorectal symptoms, but also in apical and anterior compartment support [29]. The simultaneous placement of anterior and posterior meshes, as advocated in earlier studies, utilizes similar dissection planes and operative strategies, thereby limiting additional tissue trauma and enhancing overall surgical outcomes. Given the complexity of pelvic floor dysfunctions, a multidisciplinary approach is now recognized as essential to ensure that women receive the most appropriate and individualized management. When surgery is indicated, this collaborative strategy helps determine the optimal surgical technique for each patient. The high rate of concomitant anterior SCP represents an important concomitant factor for postoperative outcome evaluation. Indeed, restoration of apical and anterior support may independently influence pelvic floor and defecatory symptoms. Therefore, the observed benefits should be interpreted in the context of combined multicompartment pelvic floor restoration.

VRP has proven to be a safe technique with a low rate of perioperative complications. In our study, only one patient required mesh removal due to infection. This finding aligns with the literature, where VRP is described as a safe procedure compared with other techniques for correcting rectal and posterior compartment prolapse [35, 36]. Mesh-related complications, including erosion, exposure, chronic infection, and the need for revision or mesh removal, may occur several years after surgery. Therefore, the mean follow-up of 18.5 months in the present study primarily reflects short- to mid-term outcomes and does not allow for definitive conclusions regarding long-term durability or late mesh-related safety.

In the multivariate analysis, none of the evaluated variables emerged as significant predictors of postoperative constipation or anal incontinence. These findings suggest that postoperative functional outcomes are likely influenced by multifactorial and patient-specific factors rather than identifiable preoperative variables. In the multivariate analysis, none of the evaluated variables emerged as significant predictors of postoperative constipation or anal incontinence. However, these analyses should be regarded as exploratory. Given the limited number of postoperative events relative to the number of candidate predictors, the study was underpowered to reliably identify independent predictors. Therefore, the absence of statistically significant associations does not exclude the presence of clinically relevant predictors.

Among the principal limitations of our study, retrospective design stands out as a potential source of bias in data collection and analysis. Symptoms other than constipation and fecal incontinence were derived from clinical documentation rather than validated questionnaires, which may introduce reporting bias. The absence of systematic validated patient-reported outcome measures (PROM)-based assessment of pelvic floor symptoms, except for the Wexner score for anorectal symptoms, limit the precision and reproducibility of functional outcome evaluation. The relatively small sample size may also restrict the generalizability of our findings to broader populations. Although enterocele and full-thickness rectal prolapse represent distinct posterior compartment entities, stratified analyses were not feasible due to sample size limitations and the very low rate of anatomical recurrence. The absence of a standardized classification system for enterocele complicates the use of consistent terminology in the medical literature, potentially hindering comparisons across studies. The absence of systematic postoperative imaging and the relatively short follow-up duration represent limitations that may lead to underestimation of late or subclinical anatomical recurrence. Nevertheless, from a clinical perspective, patient-reported symptoms and functional outcomes remain the most relevant measures of treatment success, as they reflect the actual impact of surgery on patients’ quality of life. Moreover, because anterior SCP was performed concomitantly in a large proportion of patients, functional outcomes may have been influenced by apical and anterior compartment correction, limiting causal attribution to VRP alone. The study was not powered to detect small-to-moderate differences between laparoscopic and robotic VRP, and the absence of statistically significant differences should not be interpreted as evidence of equivalence.

Despite these limitations, our study has several noteworthy strengths. It is among the first to specifically compare R-VRP and L-VRP for posterior compartment defects in a gynecological population, thereby addressing a significant gap in the current literature. Additionally, our work describes an innovative surgical approach that integrates both gynecological and colorectal expertise, utilizing similar principles and dissection techniques for the placement of anterior and posterior meshes. This multidisciplinary strategy, involving gynecologists, gastroenterologists, colorectal surgeons, radiologists, and urologists, may serve as an optimal model for the management of multicompartmental pelvic organ prolapse, with the potential to enhance functional outcomes and minimize surgical risks. A further point worth considering is the need for prospective, multicenter studies with larger cohorts and longer follow-up periods. Future prospective studies with larger cohorts, standardized PROM-based assessment, systematic imaging when clinically relevant, and longer follow-up are required to evaluate recurrence patterns, delayed mesh-related complications, and the durability of both laparoscopic and robotic VRP.

## Conclusions

In this multicentric gynecological cohort, minimally invasive VRP was associated with significant short- to mid-term anatomical and functional improvement and a low rate of perioperative complications. No statistically significant differences were observed between laparoscopic and robotic approaches; however, these findings should be interpreted cautiously given the retrospective design, baseline imbalances, high rate of concomitant procedures, limited sample size, and relatively short follow-up. Further prospective studies with larger cohorts and longer follow-up are required to clarify comparative effectiveness, long-term durability, and delayed mesh-related complications.

## Data Availability

The datasets generated and/or analyzed during the current study are not publicly available due to institutional and ethical restrictions but are available from the corresponding author on reasonable request.

## References

[1] Guzman Rojas R, Kamisan Atan I, Shek KL, Dietz HP (2016) The prevalence of abnormal posterior compartment anatomy and its association with obstructed defecation symptoms in urogynecological patients. Int Urogynecol J 27(6):939–944. 10.1007/s00192-015-2914-3 26670577 10.1007/s00192-015-2914-3

[2] Mellgren A, Johansson C, Dolk A, Anzén B, Bremmer S, Nilsson BY, Holmström B (1994) Enterocele demonstrated by defaecography is associated with other pelvic floor disorders. Int J Colorectal Dis 9(3):121–124. 10.1007/BF00290186 7814983 10.1007/BF00290186

[3] Bump RC, Norton PA (1998) Epidemiology and natural history of pelvic floor dysfunction. Obstet Gynecol Clin North Am 25(4):723–746. 10.1016/s0889-8545(05)70039-5 9921553 10.1016/s0889-8545(05)70039-5

[4] Mowat A, Maher D, Baessler K, Christmann-Schmid C, Haya N, Maher C (2018) Surgery for women with posterior compartment prolapse. Cochrane Database Syst Rev 3(3):CD012975. 10.1002/14651858.CD012975. (**PMID: 29502352; PMCID: PMC6494287.**) 29502352 10.1002/14651858.CD012975 PMC6494287

[5] Bordeianou LG, Carmichael JC, Paquette IM, Wexner S, Hull TL, Bernstein M, Keller DS, Zutshi M, Varma MG, Gurland BH, Steele SR (2018) Consensus statement of definitions for anorectal physiology testing and pelvic floor terminology (revised). Dis Colon Rectum 61(4):421–427. 10.1097/DCR.0000000000001070 29521821 10.1097/DCR.0000000000001070

[6] Haylen BT, Maher CF, Barber MD, Camargo S, Dandolu V, Digesu A, Goldman HB, Huser M, Milani AL, Moran PA, Schaer GN, Withagen MI (2016) An International Urogynecological Association (IUGA)/International Continence Society (ICS) joint report on the terminology for female pelvic organ prolapse (POP). Int Urogynecol J 27(4):655–684. 10.1007/s00192-016-3003-y 26984443 10.1007/s00192-016-3003-y

[7] Bordeianou L, Hicks CW, Kaiser AM, Alavi K, Sudan R, Wise PE (2014) Rectal prolapse: an overview of clinical features, diagnosis, and patient-specific management strategies. J Gastrointest Surg 18(5):1059–1069. 10.1007/s11605-013-2427-7 24352613 10.1007/s11605-013-2427-7

[8] Schulten SFM, Claas-Quax MJ, Weemhoff M, van Eijndhoven HW, van Leijsen SA, Vergeldt TF, IntHout J, Kluivers KB (2022) Risk factors for primary pelvic organ prolapse and prolapse recurrence: an updated systematic review and meta-analysis. Am J Obstet Gynecol 227(2):192–208. 10.1016/j.ajog.2022.04.046 35500611 10.1016/j.ajog.2022.04.046

[9] Maillard E, Henry L, Mion F, Barth X, Tissot E, Mellier G, Damon H (2008) Elytrocele with and without a history of hysterectomy (303 defecography studies). Gastroenterol Clin Biol 32(11):953–959. 10.1016/j.gcb.2008.04.036 18774666 10.1016/j.gcb.2008.04.036

[10] Lapalus MG, Henry L, Barth X, Mellier G, Gautier G, Mion F, Damon H (2004) Enterocele: clinical risk factors and association with others pelvic floor disorders (about 544 defecographies). Gynecol Obstet Fertil 32(7–8):595–600. 10.1016/j.gyobfe.2004.05.012. (**PMID: 15450257.**) 15450257 10.1016/j.gyobfe.2004.05.012

[11] Takahashi T, Yamana T, Sahara R, Iwadare J (2006) Enterocele: what is the clinical implication? Dis Colon Rectum 49:S75-81. 10.1007/s10350-006-0683-2 17106819 10.1007/s10350-006-0683-2

[12] Wijffels NA, Jones OM, Cunningham C, Bemelman WA, Lindsey I (2013) What are the symptoms of internal rectal prolapse? Colorectal Dis 15(3):368–373. 10.1111/j.1463-1318.2012.03183.x 22823279 10.1111/j.1463-1318.2012.03183.x

[13] Lin FC, Funk JT, Tiwari HA, Kalb BT, Twiss CO (2018) Dynamic pelvic magnetic resonance imaging evaluation of pelvic organ prolapse compared to physical examination findings. Urology 119:49–54. 10.1016/j.urology.2018.05.031. (**Epub 2018 Jun 23. PMID: 29944912.**) 29944912 10.1016/j.urology.2018.05.031

[14] Fitzgerald J, Richter LA (2020) The role of MRI in the diagnosis of pelvic floor disorders. Curr Urol Rep 21(7):26. 10.1007/s11934-020-00981-4. (**PMID: 32415411.**) 32415411 10.1007/s11934-020-00981-4

[15] Touchais JY, Koning E, Savoye-Collet C, Leroi AM, Denis P (2007) Place de la défécographie dans les troubles de la statique pelvienne postérieure féminine [Role of defecography in female posterior pelvic floor abnormalities]. Gynecol Obstet Fertil 35(12):1257–1263. 10.1016/j.gyobfe.2007.09.019. (**PMID: 18035577.**) 18035577 10.1016/j.gyobfe.2007.09.019

[16] Loh KC, Umanskiy K (2021) Ventral rectopexy. Clin Colon Rectal Surg 34(1):62–68. 10.1055/s-0040-1714288. (**PMID: 33536851; PMCID: PMC7843944.**) 33536851 10.1055/s-0040-1714288 PMC7843944

[17] Postillon A, Perrenot C, Germain A, Scherrer ML, Buisset C, Brunaud L, Ayav A, Bresler L (2020) Long-term outcomes of robotic ventral mesh rectopexy for external rectal prolapse. Surg Endosc 34(2):930–939. 10.1007/s00464-019-06851-6. (**Epub 2019 Jun 10. PMID: 31183789.**) 31183789 10.1007/s00464-019-06851-6

[18] Mege D, Sans A, Maignan A, Duclos J, Frasconi C, Le Huu Nho R, Pirro N, Sielezneff I (2017) Temporary successful results of ventral rectopexy for enterocele surgical correction, about 138 patients. Int J Colorectal Dis 32(11):1569–1575. 10.1007/s00384-017-2887-4. (**Epub 2017 Aug 12. PMID: 28803377.**) 28803377 10.1007/s00384-017-2887-4

[19] Vancaillie TG (1997) The role of laparoscopy in the management of pelvic floor relaxation. J Am Assoc Gynecol Laparosc 4(2):147–148. 10.1016/s1074-3804(97)80002-3. (**PMID: 9050721.**) 9050721 10.1016/s1074-3804(97)80002-3

[20] Wattiez A, Boughizane S, Alexandre F, Canis M, Mage G, Pouly JL, Bruhat MA (1995) Laparoscopic procedures for stress incontinence and prolapse. Curr Opin Obstet Gynecol 7(4):317–321 (**PMID: 7578974.**) 7578974

[21] Debbaut T, Chaoui AM, Chaoui I, Geers J, Olivier F, Schepers S, Venken R, Verbeke K, Abasbassi M (2025) Improved operative efficiency with the da Vinci Xi: a comparative study of robotics and laparoscopic ventral mesh rectopexy. J Robot Surg 19(1):676. 10.1007/s11701-025-02848-7. (**PMID: 41071235.**) 41071235 10.1007/s11701-025-02848-7

[22] Haylen BT, De Ridder D, Freeman RM, Swift SE, Berghmans B, Lee J, Monga A, Petri E, Rizk DE, Sand PK et al (2010) An International Urogynecological Association (IUGA)/International Continence Society (ICS) joint report on the terminology for female pelvic floor dysfunction. Int Urogynecol J 21:5–26. 10.1007/s00192-009-0976-9 19937315 10.1007/s00192-009-0976-9

[23] Abrams P, Khoury S (2010) International Consultation on Urological Diseases: evidence-based medicine overview of the main steps for developing and grading guideline recommendations. Neurourol Urodyn 29(1):116–118 20025028 10.1002/nau.20845

[24] van der Schans EM, Paulides TJC, Wijffels NA, Consten ECJ (2018) Management of patients with rectal prolapse: the 2017 Dutch guidelines. Tech Coloproctol 22(8):589–596. 10.1007/s10151-018-1830-1. (**Epub 2018 Aug 11. PMID: 30099626.**) 30099626 10.1007/s10151-018-1830-1

[25] Mitropoulos D, Artibani W, Biyani CS, Bjerggaard Jensen J, Rouprêt M, Truss M (2018) Validation of the Clavien-Dindo grading system in urology by the European Association of Urology guidelines ad hoc panel. Eur Urol Focus 4(4):608–613 28753862 10.1016/j.euf.2017.02.014

[26] Agachan F, Chen T, Pfeifer J et al. (1996) A constipation scoring system to simplify evaluation and management of constipated patients. Dis Colon Rectum 39(6):681–685. 10.1007/bf02056950 8646957 10.1007/BF02056950

[27] Jorge JM, Wexner SD (1993) Etiology and management of fecal incontinence. Dis Colon Rectum 36:77–97 8416784 10.1007/BF02050307

[28] D’Hoore A, Penninckx F (2006) Laparoscopic ventral recto(colpo) pexy for rectal prolapse: surgical technique and outcome for 109 patients. Surg Endosc 20(12):1919–1923. 10.1007/s00464-005-0485-y 17031741 10.1007/s00464-005-0485-y

[29] Ruffolo AF, Melocchi T, Rubod C, Kerbage Y, Campagna G, Mastrovito S, Ercoli A, Panico G, Cosson M, Lallemant M (2025) Anatomical and functional outcomes of combined ventral rectopexy and sacrocolpo/hysteropexy for multicompartment pelvic organ prolapse: a systematic review and meta-analysis. Tech Coloproctol 30(1):6. 10.1007/s10151-025-03236-x. (**PMID: 41359190; PMCID: PMC12685978**) 41359190 10.1007/s10151-025-03236-x PMC12685978

[30] Ruffolo AF, Melocchi T, Campagna G, Mastrovito S, Ercoli A, Cosson M, Lallemant M, Panico G (2025) Minimally invasive ventral rectopexy for vaginal enterocele treatment: a multicentric study. Eur J Obstet Gynecol Reprod Biol 315:114790. 10.1016/j.ejogrb.2025.114790. (**Epub 2025 Oct 24. PMID: 41151243**) 41151243 10.1016/j.ejogrb.2025.114790

[31] Campagna G, Panico G, Caramazza D, Anchora LP, Parello A, Gallucci V, Vacca L, Scambia G, Ercoli A, Ratto C (2020) Laparoscopic sacrocolpopexy plus ventral rectopexy as combined treatment for multicompartment pelvic organ prolapse. Tech Coloproctol 24(6):573–584. 10.1007/s10151-020-02199-5. (**Epub 2020 Apr 13. PMID: 32285229**) 32285229 10.1007/s10151-020-02199-5

[32] Jallad K, Ridgeway B, Paraiso MFR, Gurland B, Unger CA (2018) Long-term outcomes after ventral rectopexy with sacrocolpo- or hysteropexy for the treatment of concurrent rectal and pelvic organ prolapse. Female Pelvic Med Reconstr Surg 24(5):336–340. 10.1097/SPV.0000000000000444. (**PMID: 28657998.**) 28657998 10.1097/SPV.0000000000000444

[33] Slawik S, Soulsby R, Carter H, Payne H, Dixon AR (2008) Laparoscopic ventral rectopexy, posterior colporrhaphy and vaginal sacrocolpopexy for the treatment of recto-genital prolapse and mechanical outlet obstruction. Colorectal Dis 10(2):138–143. 10.1111/j.1463-1318.2007.01259.x. (**PMID: 17498206.,**) 17498206 10.1111/j.1463-1318.2007.01259.x

[34] Boons P, Collinson R, Cunningham C, Lindsey I (2010) Laparoscopic ventral rectopexy for external rectal prolapse improves constipation and avoids de novo constipation. Colorectal Dis 12(6):526–532. 10.1111/j.1463-1318.2009.01859.x. (**Epub 2009 Apr 10. PMID: 19486104**) 19486104 10.1111/j.1463-1318.2009.01859.x

[35] Hajibandeh S, Hajibandeh S, Arun C, Adeyemo A, McIlroy B, Peravali R (2021) Meta-analysis of laparoscopic mesh rectopexy versus posterior sutured rectopexy for management of complete rectal prolapse. Int J Colorectal Dis 36(7):1357–1366. 10.1007/s00384-021-03883-0. (**Epub 2021 Feb 23. PMID: 33624175**) 33624175 10.1007/s00384-021-03883-0

[36] van der Schans EM, Boom MA, El Moumni M, Verheijen PM, Broeders IAMJ, Consten ECJ (2022) Mesh-related complications and recurrence after ventral mesh rectopexy with synthetic versus biologic mesh: a systematic review and meta-analysis. Tech Coloproctol 26(2):85–98. 10.1007/s10151-021-02534-4. (**PMID: 34812970; PMCID: PMC8763765.**) 34812970 10.1007/s10151-021-02534-4 PMC8763765

